# Can Social Activity Participation Attenuate Aged-Related Cognitive Decline? A 12-Year Longitudinal Analysis

**DOI:** 10.1093/geroni/igaf122.2287

**Published:** 2025-12-31

**Authors:** Layla Katharine Santana, Hongdao Meng, Debra Dobbs, Yujun Liu, Angela Grippo, Jamie Mayer

**Affiliations:** University of South Florida, Tampa, Florida, United States; University of South Florida, Tampa, Florida, United States; University of South Florida, Tampa, Florida, United States; Northern Illinois University, Naperville, Illinois, United States; Northern Illinois University, DeKalb, Illinois, United States; Northern Illinois University, DeKalb, Illinois, United States

## Abstract

As populations age, the risk of cognitive decline presents significant public health challenges. While prior research highlights the role of lifestyle factors in cognitive health, less is known about the long-term protective effects of social engagement. Our previous cross-sectional study demonstrated that social participation moderates the relationship between aging and cognitive function in older adults. Building upon these findings, this study investigates how social participation influences cognitive trajectories in aging populations using data from the Health and Retirement Study (HRS) from 2008 to 2020. The sample included 6,109 adults aged 50 and older. Cognitive function scores were assessed across four waves (2008, 2012, 2016, 2020), and social activity participation was measured using a validated 10-point scale. A linear mixed-effects regression model, implemented in Stata, was used to estimate changes in cognition while accounting for key sociodemographic and health-related covariates. The mean participant age was 65.4 years (SD = 8.87); 57.8% were female, and 83.8% were White. Findings revealed that higher social activity participation predicted better cognitive function, with particularly beneficial effects observed in older age. Notably, the interaction effect indicated that increasing social engagement over time may slow cognitive decline. By examining these dynamics longitudinally, we provide deeper insight into the long-term protective effects of social participation. These findings can inform targeted interventions and policies aimed at supporting cognitive resilience in aging populations. Addressing barriers to social engagement remains critical for enhancing not only social well-being but also cognitive health among older adults.

